# Evaluation of behavioral changes and subjective distress after exposure to coercive inpatient interventions

**DOI:** 10.1186/1471-244X-12-54

**Published:** 2012-05-30

**Authors:** Irina Georgieva, Cornelis L Mulder, Richard Whittington

**Affiliations:** 1 Department of Psychiatry, Erasmus, MC, Rotterdam, 3000, CA, PO Box 2040, The Netherlands; 2 Mental Health Center Western North-Brabant, Halsteren, The Netherlands; 3 Research Center O3, Erasmus MC, Rotterdam, The Netherlands; 4 Department of Psychiatry, Bavo-Europoort and Municipal Health Center Rotterdam, Rotterdam, The Netherlands; 5 Institute of Psychology, Health and Society, University of Liverpool & Honorary Research Fellow, Mersey Care NHS Trust, Liverpool, United kingdom

**Keywords:** Coercive measures, Seclusion, Mechanical restraint, Involuntary medication, Coercion

## Abstract

**Background:**

There is a lack of evidence to underpin decisions on what constitutes the most effective and least restrictive form of coercive intervention when responding to violent behavior. Therefore we compared ratings of effectiveness and subjective distress by 125 inpatients across four types of coercive interventions.

**Methods:**

Effectiveness was assessed through ratings of patient behavior immediately after exposure to a coercive measure and 24 h later. Subjective distress was examined using the Coercion Experience Scale at debriefing. Regression analyses were performed to compare these outcome variables across the four types of coercive interventions.

**Results:**

Using univariate statistics, no significant differences in effectiveness and subjective distress were found between the groups, except that patients who were involuntarily medicated experienced significant less isolation during the measure than patients who underwent combined measures. However, when controlling for the effect of demographic and clinical characteristics, significant differences on subjective distress between the groups emerged: involuntary medication was experienced as the least distressing overall and least humiliating, caused less physical adverse effects and less sense of isolation. Combined coercive interventions, regardless of the type, caused significantly more physical adverse effects and feelings of isolation than individual interventions.

**Conclusions:**

In the absence of information on individual patient preferences, involuntary medication may be more justified than seclusion and mechanical restraint as a coercive intervention. Use of multiple interventions requires significant justification given their association with significant distress.

## Background

Coercive interventions such as seclusion, involuntary medication and mechanical restraint are common methods for managing violent behavior during psychiatric hospitalization. Even though they are intended to protect patients and those around them, they are highly controversial, because they restrict freedom and are used against a patient’s will. They are even more problematic when used in combination – for example, when seclusion is combined with mechanical restraint. They can also be extremely traumatic [1], causing physical and psychological damage to patient and staff alike [2]. As a result, practitioners contemplating their use are confronted with a serious ethical and professional dilemma.

It is widely accepted in mental health services around the world that coercion is a last resort and should be proportionate to the degree of threat being faced [3-5]. For this reason, it should always be decided whether its possible dangers are considerably outweighed by the likely benefits to the patient and others. Similarly, the principle of subsidiarity requires that the intervention is justifiable only if no other, less coercive, interventions are available to deal with the imminent threat [6]. In other words, an individualized approach is required in which the most effective and least damaging intervention for the particular person is established.

To make such a judgement, mental health professionals need to have substantial knowledge of the effectiveness and harmfulness of the various coercive interventions. Unfortunately, there is not enough evidence on the relative effectiveness and harm of specific interventions such as seclusion or restraint [7]. Recently two studies have been published, comparing the effectiveness and impact of seclusion and mechanical restraint [8,9]. Despite their excellent methodological designs (i.g. randomized controlled trials) studies had excellent methodological design, their relevance for clinicians in constructing a hierarchy for the use of coercive interventions is limited in comparison to this study, because their scope is restricted to two interventions (i.e. seclusion and mechanical restraint). In addition both studies found no significant differences between the groups in patients’ experienced coercion or satisfaction with care. Other studies on this topic tend to focus on staff and patient attitudes rather than actual experiences [10-13]. As attitudes are likely to be influenced by previous coercive experiences [14] and by traditions and culture [15], studying them is more likely to explain differences in coercive practices between institutions [16] and between countries [17,18] than to provide a basis for clinical decisions.

To obtain information which can help clinicians to apply the proportionality and subsidiarity principles, it is not just attitudes that should be compared, but coercive interventions. Ideally, such comparisons should use validated assessment instruments in “real life” settings. Directly and one-to-one, they should contrast individual interventions (such as seclusion vs. involuntary medication) and their combinations (such as seclusion and mechanical restraint vs. seclusion alone). Here we report such an “in vivo” study using formal assessments.

A further issue in any decision to use coercion is the balance of therapeutic and safety factors. The international literature has still found no consensus on whether coercive interventions are simply safety interventions whose aim is not to provide therapeutic value but simply to reduce the imminent risk of danger to the individual or others, whether they are counter-therapeutic treatment failures [19], or whether they are therapeutic interventions that also improve a patient’s psychological functioning. We therefore evaluated both aspects, measuring changes in aggression and uncooperativeness on the one hand, and changes in psychological functioning and insight on the other.

### Aims of the study

To compare ratings of effectiveness and subjective distress with respect to the following: 1) different types of coercive methods, especially seclusion versus involuntary medication; and 2) individual versus combined measures.

## Methods

### Hospital characteristics and study design

Data for this study were collected from November 2007 until October 2010 in an acute ward in a psychiatric hospital that provides care to a catchment area of around 276,000 people in the south-western Netherlands.

We studied all patients who underwent coercion during the research period. The study used a prospective design that examined the relationships between independent variables (type of coercive intervention, demographic and clinical characteristics) and two dependent variables (effectiveness and subjective distress). The index intervention for the study was the first coercive intervention after admission. Although there may have been other coercive interventions during the stay in the ward, our evaluation in this study is based only on the index intervention.

The research was approved by the local Medical Ethical Committee, which waived the requirements for informed consent because the research involved no risks to the patients, and because data were being collected as part of a policy-control procedure.

### Procedure

#### Definitions of coercive interventions

The Netherlands’ Mental Health Act ranks five coercive interventions equally for management of acute danger – seclusion, involuntary medication, isolation, mechanical restraint and forced feeding – an individualized approach requires that the least harmful and most effective intervention is applied.Seclusion was defined as the placement of a patient in a locked room from which free exit was denied for a fixed period of time.

Involuntary medication was defined as the administration of a rapid tranquilizer without the consent of the patient, and with or without manual restraint. Rapid tranquillization involved the oral or intramuscular administration of a combination of haloperidol and promethazine, or lorazepam to achieve rapid, short-term behavioural control of any extreme agitation, aggression or potentially violent behaviour that placed the individual and those around them at risk. Initially, 10 mg haloperidol and 100 mg promethazine, or lorazepam 2½-5 mg was offered as oral medication to the agitated patients with psychotic or non-psychotic symptoms, respectively. Nevertheless, in some situations patients refused to take the medication orally, so IM medication (5 mg haloperidol and 50 mg promethazine or 2½-5 mg lorazepam) was used. Due to the coercive nature of the setting, administration of “as required” medication during a period of seclusion was also counted as involuntary medication, regardless of patient consent at the time.

Mechanical restraint was defined as the application of any mechanical device which limited the patient’s movement, physical activity, or normal access to his or her body.

For the purpose of this study, combination of coercive measures involved any use of more than one of the interventions specified above. In practice, there were two types of combined intervention: seclusion plus medication, and seclusion plus mechanical restraint (including a few cases in which involuntary medication was also used).

#### Independent variables

Data on gender, age, and voluntary/involuntary admission status were collected from patients’ records. Past coercive experiences, ethnicity and marital status were established by interviewing patients directly after admission. DSM-IV diagnoses were generated by the psychiatrist on the ward and obtained form the patients’ chart. Data on the type and duration of the restrictive measures were extracted from the hospital database after the episode had finished. The staff assessed the level of coercion/pressure they had applied at the beginning of every coercive intervention on a scale from 0 to 10. A higher score signifies more coercion.

#### Dependent variables

Effectiveness was operationalized in four ways: 1) psychological functioning, 2) insight into the illness, 3) uncooperativeness with treatment, and 4) aggressive behaviour. These variables were twice rated by nurses who had been trained in the use of the respective instruments (see below). The first rating was made immediately after the patients had begun their exposure to the restrictive measure or measures. The second was made 24 h later. Analyses were based on a change score, i.e. on changes in these four dimensions between the two time points. As some interventions lasted longer than 24 h – especially seclusion (29.5% of the incidents) – the second assessment may have taken place while the patient was still subjected to it.

During a standard debriefing procedure in the week that followed the end of the intervention, subjective distress was examined by assessing the patients’ experience with the coercive measure or measures.

##### Instruments for assessing effectiveness

The patient’s general wellbeing and level of functioning was assessed using the short version of the Kennedy Axis V [20]. This consists of four domains: 1) psychological impairment 2) social skills 3) violence, and 4) activities of daily living (ADL) and occupational skills, each rated from 10 to 100. A higher score reflects better functioning. The mean score of these domains was used to derive the global assessment of functioning (GAF) score used in the study.

The level of uncooperativeness and lack of judgment and insight at admission were determined on the basis of items G8 and G12 of the Positive and Negative Syndrome Scale (PANSS) [21]. Each item was rated on a scale from 1 (absent) to 7 (extreme).

Aggression was assessed using the Social Dysfunction and Aggression Scale (SDAS) [22], which contains 11 items scored from 0 (not present) to 4 (extremely severe). As well as nine items covering interpersonal (other-directed) aggression (i.e. non-directed verbal aggressiveness, directed verbal aggressiveness, irritability, negativism, dysphoric mood, socially disturbed behaviour, physical violence to staff, physical violence to others, and physical violence to things), it consists of two items covering self-harm (i.e. suicidal behaviour vs. self-injurious behaviour). The reliability of this scale is high (interclass coefficient: .97, Cronbach’s a: .79) [22]. The validity of the SDAS is high as well: the sum-scores of the scales MOAS [23], SDAS, and SOAS [24] correlate highly (r between .78 and .91) [25].

##### Instrument for assessing subjective distress

During the debriefing that followed the end of a coercive intervention, patients filled in the Coercion Experience Scale (CES) [26], an instrument to measure the psychological and physical impact of coercive interventions in mental health settings. The reliability and validity of its psychometric properties are satisfactory, as follows: Cronbach alpha of the CES scale ranged from .67 to .93, while the convergent and discriminant validity yielded respectively: r = .79 (p < .001), and r = .38 (p < .001) [26]. The questionnaire consists of six factors: “humiliation“(14 items, e.g. “dignity taken away”); “physically adverse effects” (4 items, e.g. “pain”); “separation” (2 items, e.g. “restrictions of interpersonal contact”); “negative environment” (5 items, e.g. “fear of not getting enough air”); “fear” (2 items, e.g. “afraid to die”); and “coercion“(2 items, e.g. “the applied coercion was…”). Each item is assessed on a Likert-Scale that provides scope to indicate the degree to which the coercive method was stressful (not at all/mildly/moderate/severely/extreme) or how it had been experienced (acceptable/uncomfortable/unpleasant/very  unpleasant/extremely unpleasant). In addition, a visual-analogue-scale (VAS) was used to measure the overall burden of the coercive measure.

Since the original questionnaire was developed specifically to compare seclusion with mechanical restraint, we added three items to cover the subjective distress that had been experienced upon the administration of involuntary medication: “I was held by staff members”, “I got medication against my will” and “My functioning was hindered by side-effects of the medication”. We analyzed these three items by adding them to the total score of the CES. They were also included in the revised edition of the questionnaire by the author of the scale.

### Statistics

To compare patients’ socio-demographic and clinical details, we used Chi square analyses and Anova F tests in four groups based on the coercive interventions they had experienced: (1) seclusion alone, (2) medication alone, (3) seclusion and medication combined, and (4) seclusion and mechanical restraint combined. To achieve normal distribution, some variables were logarithmically transformed. If the data were still skewed, non-parametric tests (e.g. Wallis & Mann Whitney U) were used.

Multiple linear regression analyses were then used to explore associations between the two main dependent variables: effectiveness over 24 h and the subjective distress of the episode as assessed at its conclusion on the one hand and the type of coercive intervention(s) on the other, whilst controlling for the effect of other independent variables and baseline scores. The four types of coercive interventions were entered into the regression analyses using combined seclusion and mechanical restraint as a reference group. In addition, separate regression analyses were conducted to compare seclusion alone (reference group) versus medication alone, and individual interventions (reference group) versus combined interventions. All other independent variables were entered using the stepwise method.

## Results

### Descriptives and univariate analyses

In total, 125 patients underwent coercion during the research period. Between a third and three-quarters were male (65%), single (75%), of Dutch origin (72%), and had been admitted involuntarily (69%). The average age was thirty seven years (SD = 13). Most of the patients suffered from a psychotic disorder (39%), followed by a mood disorder (33%), addiction (drugs or alcohol; 26%), personality disorder (12%), and post-traumatic stress disorder (PTSD) (4%). Forty-six (37%) patients reported having been coerced during previous periods of hospitalization. During the study, 52 patients received involuntary medication; in over half the cases, this was administered orally (57%).

Table 1 reports descriptive data on all variables across the four intervention groups. Combined interventions were used most among patients with a psychotic disorder, which was therefore the diagnostic criterion we used to dichotomize the data for further analyses (psychotic disorder: yes or no). Those subjected to seclusion in combination with medication were significantly more likely to have been subjected to coercive experiences during previous hospitalizations. Seclusion episodes combined with mechanical restraint were (non-significantly) longer than those combined with medication, or those in which seclusion was used on its own. This is probably because patients subjected to seclusion and mechanical restraint were significantly less well (lower GAF score), had less insight into their illness, or were more uncooperative at the start of the intervention than the patients who were secluded only.

**Table 1:** Patients’ demographic and clinical characteristics divided in four groups according to the applied coercive intervention(s) during the first 24 h

**Demographic and Clinical characteristics**						
		**Coercive experience**				
**Variable**	N*	**Group 1** **Seclusion only**	**Group 2** **Involuntary medication only**	**Group 3** **Combined** **Seclusion & medication**	**Group 4** **Combined Seclusion & mechanical restraint**	**P**
		**N = 62**	**N = 18**	**N = 34**	N = 11**		
		**N (%)/Mean (SD)**	**N (%)/Mean (SD)**	**N (%)/Mean (SD)**	**N (%)/Mean (SD)**		
Male gender	125	36(58%)	11(61%)	24(71%)	10(91%)	0.2	
Mean age	125	36(12)	38(13)	39(13)	35(15)	0.6	
Married status: single	118	45(48%)	15(16%)	25(27%)	9 (10%)	0.7	
**Ethnicity**							
1^st^ & 2nd generation immigrants	120	13(22%)	5(28%)	7(22%)	3(27%)	0.9	
**Legal status upon admission**							
Involuntary commitment	119	37(65%)	12(67%)	24(73%)	9(82%)	0.7	
Coercive experience during previous admissions	106	19(36%)	2(13%)	17(63%)	5(46%)	**0.01¹**	
Patients and their diagnosis***	119						
Psychotic disorder	46	16(27%)	7(39%)	17(53%)	6(60%)	**0.045**^**2**^	
Mood disorder	39	20(34%)	6(33%)	12(38%)	1(10%)	0.4	
Personality disorder	14	5(9%)	6(33%)	3(9%)	0	-	
Addiction	31	19(32%)	5(28%)	4(13%)	3(30%)	0.2	
PTSD	5	3(5%)	0	2(6%)	0	-	
Oral (versus intramuscular) administration of medication	52	X	14(78%)	12(43%)	2(50%)	0.08	
Level of coercion at the start of the measure	115	3.6(3)	3(3)	4.8(3)	5.8(3.8)	0.08	
Mean duration seclusion episode in hours****	105	21(31)	X	31(38)	65(67)	0.13	
**Mean score GAF**							
Pre-measurement	125	44 (9)	41 (9)	41 (11)	35 (12)	**0.03**^**3**^	
Post-measurement	122	60(16)	54(16)	54(14)	54(20)	0.2	
Change score****	122	16(14)	12(15)	14(15)	19(20)	0.3	
**Mean score Uncooperativeness (PANSS)**							
Pre-measurement*****	125	5(1.5)	5(1.5)	6(1.4)	6(1)	**0.001**^**4**^	
Post-measurement	119	3(1)	4(2)	3(2)	4(2)	0.4	
Change score	119	−1.7(1.7)	−1(1.9)	−2.5(2)	−2(2)	0.1	
**Mean score Lack of judgment and Insight (PANSS)**							
Pre-measurement*****	125	5(1.5)	5(1.5)	6(1.6)	6(0.9)	**0.001**^**5**^	
Post-measurement	119	3.5(1)	4.3(1.9)	4.2(1.7)	4.8(1.6)	0.03	
Change score	119	−1.2(1.5)	-.9(1.7)	−1.6(1.7)	−1.4(1.8)	0.5	
**Mean score SDAS**							
Pre-measurement	125	18(9)	18(7)	21(8)	22(10)	0.2	
Post-measurement****	119	7(7)	11(9)	8(8)	10(12)	0.5	
Change score	119	−11(9)	−7(9)	−13(9)	−14(9)	0.1	

* The *n* and the percentage of respondents vary across the variables, because some of the clinical files were incomplete.

** This includes one patient who received extra involuntary medication and 3 patients who received prn medication.

*** 20% of the patients had more than one diagnose.

**** Analyses were conducted with the logarithmic transformed scores to normalize the distribution.

***** Non-parametric tests (Kruskal Wallis & Mann Whitney U) were used.

Post-hoc analyses:

¹ Group 3 differs significantly from group 1 & group 2.

^2^ Group 1 differs significantly from group 3.

^3^ Group 1 differs significantly from group 4.

^4^ Group 1 & 2 differs significantly from group 3 & group 4.

^5^ Group 1 differs significantly from group 3 & group 4; group 2 differs significantly from group 4.

In terms of effectiveness, Table 1 also shows clearly that all groups improved 24 h after undergoing the coercive intervention (GAF score (t = −11.4, df = 121, p. <001); insight into the illness (Wilcoxon, Z = −6.9, p < .001); reduction in uncooperativeness (Wilcoxon, Z = −7.9, p < .001); and reduction in aggression (Wilcoxon, Z = −8.5, p < .001). However, there were no significant differences in these change scores between the groups.

**Figure 1:**
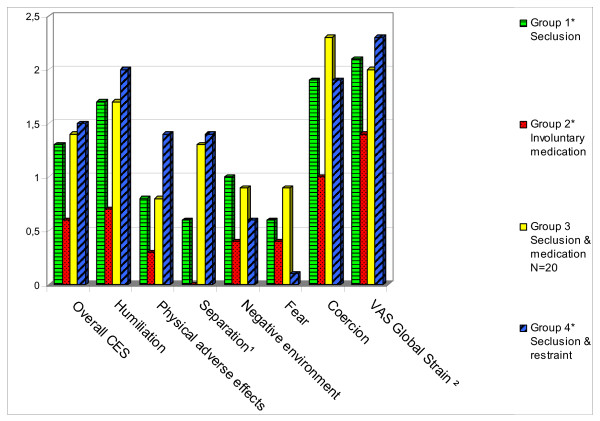
**Subjective distress compared between four types of coercive interventions on the Coercion Experience Scale (CES).** ¹Group 2 differs significantly from group 3 & group 4 on Separation. ² The mean values of VAS Global Strain were divided by 30 to stay in proportion with the rest of the scales. * The number of respondents varies in a range between: 44 and 46 (Group 1); 9 and 11 (Group 2); 8 and 9 (Group 4). ** Higher score indicates more psychological and physical burden.

Figure 1 illustrates differences in subjective distress across the four intervention groups. Because some patients were subjected to an additional coercive measure (or measures) 24 h after the start of the first coercive intervention, fewer respondents were included with regard to this outcome variable. It was also the case that 40% of the coerced patients refused to fill in the Coercion Experience Scale (CES), or were discharged before debriefing. These patients had a significantly lower GAF score and were more uncooperative (p < .05) at the respective post-measurements.

There was no significant variation between the groups in CES total and factor scores, apart from Factor 3 (Separation), which was rated significantly higher by those who had been subjected to combined interventions than by those who had been medicated only.

### Results from multiple regression analyses

Tables 2, 3 and 4 report findings from the regression analyses, which show only regression models with at least one significant predictor (beyond baseline scores of the dependent variables). It was shown by comparison of group 1 (seclusion), group 2 (involuntary medication) and group 3 (seclusion combined with involuntary medication) with group 4 (seclusion combined with mechanical restraint) that type of coercive intervention did not predict any aspects of effectiveness (Table 2). However, lower psychological and physical burden (including overall CES, and the factors humiliation, physically adverse events, and feelings of separation) was significantly associated with the use of involuntary medication as compared to seclusion combined with mechanical restraint, after controlling for demographic and clinical variables.

**Table 2:** Results of regression analyses investigating the associations between effectiveness and subjective distress (CES), and type of coercive interventions, controlling for demographical and clinical variables (patients who experienced seclusion and mechanical restraint constituted the reference group; only models with at least one significant predictor beyond baseline scores are reported here)

**Dependent variables**	**Change scores Effectiveness**			**Subjective distress : Mean scores Coercion Experience Scale (CES)**						
	**SDAS**	**Uncooperative-ness**	**Lack of insight into the illness**	**Total score CES**	**Factor Humiliation**	**Factor Physical adverse effects**	**Factor Separation**	**Factor Negative environment**	**Factor Fear**	**Factor Coercion**
**Independent variables1**	df(6;92)	df(5;93)	df(5;93)	df(5;68)	df(5;68)	df(6;68)	df(5;71)	df(6;68)	df(5;69)	df(4;73)
	R² = 0.4***	R² = 0.4***	R² = 0.3***	R² = 0.3***	R² = 0.2**	R² = 0.4****	R² = 0.26**	R² = 0.34***	R² = 0.2**	R² = 0.13*
	Unstandardized coefficients	Unstandardized coefficients	Unstandardized coefficients	Unstandardized coefficients	Unstandardized coefficients	Unstandardized coefficients	Unstandardized coefficients	Unstandardized coefficients	Unstandardized coefficients	Unstandardized coefficients
Seclusion	1.9	−0.2	−0.5	−0.5	−0.7	−0.9**	−1.2*	0.2	0.6	0.2
Involuntary medication	6.1	0.2	−0.003	−1**	−1.5**	−1.4***	−1.8***	−0.3	0.5	−0.5
Seclusion & medication	-.08	−0.5	−0.5	−0.3	−0.6	−0.8*	−0.5	0.3	0.8	0.4
Female gender	n.s.	n.s.	n.s.	0.6***	0.6*	0.9***	0.7**	0.8***	0.6*	n.s.
Age	n.s.	n.s.	n.s.	−0.02**	−0.02*	−0.02*	n.s.	−0.02**	n.s.	n.s.
Married status	n.s.	n.s.	n.s.	n.s.	n.s.	−0.3*	−0.6*	n.s.	n.s.	n.s.
Voluntary status at admission	n.s.	n.s.	n.s.	n.s	n.s.	n.s.	n.s.	−0.5**	n.s.	n.s.
Coercive experience during previous admissions	3.8*	n.s.	n.s.	n.s.	n.s.	n.s.	n.s.	n.s.	n.s.	n.s.
Psychotic disorder	3.8*	0.9**	0.7*	n.s.	n.s.	n.s.	n.s.	n.s.	n.s.	n.s.
Pressure applied from the staff at the start of the measure	n.s.	n.s.	n.s.	n.s.	n.s.	n.s.	n.s.	n.s.	0.09**	0.1*
Baseline score of SDAS, Uncooperativeness and lack of insight, respectively	−0.7***	−0.7***	−0.6***	N/A²	N/A	N/A	N/A	N/A	N/A	N/A

* significant at the 0.05 level; ** significant at the 0.01 level; *** significant at the 0.001 level.

¹ Ethnical minority was excluded from all stepwise regression analyses; ²*N/A* not applicable.

**Table 3:** Results from regression analyses investigating the associations between effectiveness and subjective distress (CES) of patients experienced involuntary medication and seclusion with other clinical and demographical variables (codes: involuntary medication (1); seclusion (0); only models with at least one significant predictor beyond baseline scores, are reported here)

**Dependent variables**	**Change scores Effectiveness**	**Subjective distress : Mean scores Coercion Experience Scale (CES)**					
	**Uncooperativeness**	**Total score CES**	**Factor** **Humiliation**	**Factor** **Physical adverse effects**	**Factor** **Negative environment**	**Factor** **Coercion**	**VAS Global Strain**
**Independent variables1**	df(3;64)	df(4;48)	df(1;51)	df(3;50)	df(3;50)	df(2;55)	df(1;50)
	R² = 0.4***	R² = 0.3***	R² = 0.1	R² = 0.3	R² = 0.3	R² = 0.13*	R² = 0.08**
	Unstandardized coefficients	Unstandardized coefficients	Unstandardized coefficients	Unstandardized coefficients	Unstandardized coefficients	Unstandardized coefficients	Unstandardized coefficients
Involuntary medication	0.4	−0.6*	−0.9*	−0.4	−0.6*	−0.7	−21*
Female gender	n.s.	0.6*	n.s.	0.7**	0.7**	n.s.	n.s.
Age	n.s.	−0.02*	n.s.	−0.03**	n.s.	n.s.	n.s.
Married status	n.s.	n.s	n.s.	n.s.	−0.7**	n.s.	n.s.
Psychotic disorder	0.9*	n.s.	n.s.	n.s.	n.s.	n.s.	n.s.
Pressure applied from the staff at the start of the measure	n.s.	0.7*	n.s.	n.s.	n.s.	0.12*	n.s.
Baseline score of Uncooperativeness	−0.7***	N/A²	N/A	N/A	N/A	N/A	N/A

* significant at the 0.05 level; ** significant at the 0.01 level; *** significant at the 0.001 level

; 1 Independent variables excluded from all stepwise regression analyses: ethnical minority, voluntary status & coercive experience during previous admissions; ² *N/A* not applicable.

**Table 4:** Results from regression analyses investigating the associations between effectiveness and subjective distress (CES) of patients experienced individual and combined interventions with other clinical and demographical variables (codes: combined interventions (1); individual interventions (0); only models with at least one significant predictor beyond baseline scores, are reported here)

**Dependent variables**	**Change score Effectiveness**			**Subjective distress : Mean scores Coercion Experience Scale (CES)**						
	**SDAS**	**Uncooperative-ness**	**Lack of insight into the illness**	**Total score**	**Factor** **Humiliation**	**Factor** **Physical adverse effects**	**Factor** **Separation**	**Factor** **Negative environment**	**Factor** **Fear**	**Factor** **Coercion**
**Independent variables1**	df(4;94)	df(3;95)	df(3;95)	df(4;69)	df(3;70)	df(3;71)	df(2;74)	df(4;70)	df(3;71)	df(2;75)
	R² = 0.4***	R² = 0.4***	R² = 0.3***	R² = 0.26***	R² = 0.13*	R² = 0.3***	R² = 0.18**	R² = 0.3***	R² = 0.16**	R² = 0.09*
	Unstandardized coefficients	Unstandardized coefficients	Unstandardized coefficients	Unstandardized coefficients	Unstandardized coefficients	Unstandardized coefficients	Unstandardized coefficients	Unstandardized coefficients	Unstandardized coefficients	Unstandardized coefficients
Combination	−2.9	−0.4	−0.02	0.3	0.4	0.4*	0.9***	0.07	0.07	.3
Female gender	n.s.	n.s.	n.s.	0.6**	0.6*	0.8***	0.7*	0.8***	0.6**	n.s.
Age	n.s.	n.s.	n.s.	−0.02**	−0.02*	−0.03***	n.s.	−0.02**	n.s.	n.s.
Voluntary status at admission	n.s.	n.s.	n.s.	n.s	n.s.	n.s.	n.s.	−0.5*	n.s.	n.s.
Coercive experience during previous admissions	3.3*	n.s.	n.s.	n.s.	n.s.	n.s.	n.s.	n.s.	n.s.	n.s.
Psychotic disorder	4*	0.97**	0.7*	n.s.	n.s.	n.s.	n.s.	n.s.	n.s.	n.s.
Pressure applied from the staff at the start of the measure	n.s.	n.s.	n.s.	0.06*	n.s.	n.s.	n.s.	n.s.	0.09*	0.1*
Baseline score of SDAS, uncooperativeness and lack of insight, respectively	−0.7***	−0.7***	−0.6***	N/A²	N/A	N/A	N/A	N/A	N/A	N/A

* significant at the 0.05 level; ** significant at the 0.01 level; *** significant at the 0.001 level

¹ Independent variables excluded from all stepwise regression analyses: married status, ethnical minority & coercive experience during previous admissions; ² *N/A* not applicable.

With regard to their association with lower levels of physically adverse events, all methods were significantly better than seclusion plus mechanical restraint. Similarly, coercive experience during previous periods of hospitalization, and psychotic disorder were positively associated with changes in effectiveness scores, while increased levels of subjective distress were significantly associated with female gender, involuntary admission status, pressure applied by the staff at the start of the measure, lower age, and unmarried status.

In subsequent analyses we compared seclusion alone with involuntary medication alone (Table 3). These did not differ with regard to predicting relative changes in GAF, insight, uncooperativeness and aggression. Involuntary medication was a significant predictor of lower CES total score, humiliation, unpleasant environment, and lower global strain. Gender, age, marital status and pressure applied by the staff again emerged as significant predictors of various aspects of psychological and physical burden.

Further, comparison of any combined coercive intervention (“seclusion plus”) with any singular intervention (seclusion alone and involuntary medication alone), showed that combined measures (Table 4) were associated with higher subjective distress, more specifically with causing more feelings of separation and more physically adverse effects. Pressure applied by the staff at the beginning of the measure significantly increased feelings of fear and coercion during the intervention.

## Discussion

This study set out to investigate the relative effectiveness of four types of coercive interventions used by the mental health services, and the relative psychological and physical burdens these interventions imposed on patients. The study, which used structured and validated assessment tools completed by trained staff at two time points, also sought to assess the patient’s perspective through interviews conducted after the coercive event.

By using change scores, it is possible to identify improvement or deterioration in mental state after the implementation of a coercive intervention. It is noticeable that mental health (GAF) and behavior (SDAS, PANSS) improved in all four groups irrespective of the measure to which they had been exposed. While it is possible for reductions in conflict behaviors such as aggression (SDAS) and uncooperativeness (PANSS) to reflect submission to the authority and power of the staff that acts coercively, more notable were the improvements in wellbeing (GAF) and insight. Nevertheless all such ratings are at risk of contamination, as they were completed by staff who were not blind to the allocated intervention, and who were likely to have an investment in reporting improvements. It is also possible that there would have been equal or even better improvements in wellbeing and insight if the coercive interventions had not been used. Only a controlled research design would be able to establish this relationship, which is difficult to apply when violent behavior needs to be managed.

Although testing in both the univariate and multivariate analyses did not enable us to find any significant differences in effectiveness between the coercive interventions, there were differences between the groups with regard to the change scores for psychological functioning (change scores for GAF and insight into the illness) and reduction of imminent danger (change scores for aggression and uncooperativeness) (see Table 1). This suggests that differences in effectiveness might become significant with a larger sample.

Our study raises a number of key issues. Firstly, it provides evidence that, when all else fails and a patient’s preference has not been previously recorded, involuntary medication should be the treatment of choice if a coercive intervention is unavoidable. In the first instance, the patient’s unequivocal consent to the oral route of administration is recommended, not only because oral administration is experienced as less coercive [27] but also because it manages acute agitation just as effectively as an intramuscular formulation [28].

When seclusion was not part of the coercive intervention, patients in our study who received medication alone experienced less isolation. After controlling for the effect of other variables, involuntary medication emerged as significantly associated with lower burden in more aspects of CES (i.e. overall CES, humiliation, and physically adverse events) than seclusion with or without restraint was. Medicated patients also reported substantially less global strain than patients who had been secluded only.

Conversely, we found that, as reported earlier [29], subjecting the patient to a combination of seclusion and mechanical restraint is highly aversive and should be the least preferred option. As 9% of our sample was subjected to this highly intrusive intervention, it is by no means uncommon – and, given the availability of the less intrusive interventions examined here, could also be said to risk contravening the well-established principle of proportionality. If seclusion episodes were combined with mechanical restraint, they were more than twice as long as seclusion alone or seclusion combined with medication. Although the restraint group sample was small (n = 11), there is no evidence that, in terms of aggression and psychological functioning, the restrained group were more disturbed than the secluded and medicated group at the onset of the intervention.

Further, combining seclusion with mechanical restraint was not significantly more effective in improving psychological functioning or reducing aggression than the rest of the restrictive measures were. However, all three other types of intervention as measured by some of the CES’s subscales were associated with lower burden; this further indicates the psychological costs to the patient of being restrained and secluded. These findings are in line with the recommendation of the Council of Europe as stated in the White Paper: “seclusion and mechanical or other means of restraint for prolonged periods should be resorted to only in exceptional cases” [3].

By the same token, there is evidence that the combination of coercive interventions should be avoided – regardless of the types being combined. The principle of proportionality indicates that, because combined interventions were not more effective, single interventions should be used – and our findings indicate that these single interventions should preferably consist of medication.

It is also clear that different groups of patients react differently to the coercive situation. This variation amongst gender and age groups in terms of attitudes to coercive measures has been observed elsewhere [10]. Our own study produced evidence that women and younger people reported that they had experienced coercive interventions as more burdensome – something staff should be aware of when deciding on implementing coercive interventions. This higher reported burden may of course reflect a willingness to report feelings of vulnerability, but it may also reflect not just women’s lower average tolerance thresholds for painful stimuli [30], but also, as a socially influenced gender-based characteristic, their greater emotional responsiveness [31]. While no decision to coerce should be taken lightly in this context, it seems that decisions to coerce women should be considered particularly carefully.

At the start of the coercive interventions, nursing staff should also use as little pressure as possible, because it may increase patients’ feelings of fear and coercion during the intervention. This may aggravate their condition: previous research has provided strong evidence that anxiety is related to the occurrence of persecutory delusions [32], paranoia and hallucinations [33]. Such interventions may thus end up counteracting the main therapeutic goal of psychiatric admission, which is to reduce symptoms and bizarre behaviours – although in this study we have also noted the general improvement in psychological functioning brought about by the coercive intervention(s).

In addition to this, increased perceived coercion might lead a patient to disengage from psychiatric services. It can also seriously damage the therapeutic relationship [34]. In order to facilitate effective communication and aid the patients’ recovery, patients should be encouraged to participate and negotiate in decision-making on their own care [35]. Increased feelings of coercion, humiliation, physically adverse effects and fear can also cause serious long-lasting adverse effects like retraumatization [36] and PTSD.

### Study limitations

While this is the first study yielding evidence that involuntary medication is less distressing for patients than seclusion or restraint by exploring actual coercive experiences, we must acknowledge a number of limitations. Firstly, 40% of the coerced patients refused to fill in the CES or were discharged before debriefing. Although a response rate of 60% has been described as good in an acute setting with difficult-to-recruit patients [37], the non-respondents were significantly less cooperative and had lower GAF, so it is possible that the most seriously ill and traumatized patients were unable to participate, or refused to, making the CES scores here an underestimate of the real burden.

Secondly, patients were interviewed by the nursing staff and not by an independent researcher. Again, it is therefore possible that patients underreported the intensity of their experience to avoid challenging the staff.

Thirdly, a randomized clinical design and a larger sample size would clearly be preferable for establishing the effectiveness of these interventions [38]. But such a design is extremely difficult to implement for this particular question, and samples are difficult to recruit. Although patients were not randomly allocated to the interventions groups and patients’ condition differed significantly between some of the groups at the start of the coercive interventions, we controlled for these baseline differences in the analyses.

Fourthly, the setting here in a single Dutch mental health unit makes widespread generalizability to other services and national policy contexts difficult.

Fifthly, we could not subdivide patients according to whether they received oral or intramuscular medication because of the small sample size.

Finally, we should add that the scope of our study was limited to the coercive measures that are used most often in the Netherlands, and that we did not evaluate the effects of other restrictive interventions such as physical restraint, continuous observation, or time-out. Ideally, the next step in this field would be an international multi-centre study conducted in way that assessed differences in a broader range of coercive practices and patients’ responses.

## Conclusions

In the absence of information on individual patient preferences, evidence here suggests that seclusion and mechanical restraint are less justified than involuntary medication as a coercive intervention. Besides that, use of multiple interventions requires significant justification given their association with significant distress.

## Competing interests

The authors declare that they have no competing interests.

## Authors’ contributions

CLM and IG developed the study design, IG supervised the data collection and analyzed the data, RW and IG drafted the manuscript. All authors revised the manuscript critically for important intellectual content and approved the final version to be published.

## Pre-publication history

The pre-publication history for this paper can be accessed here:

http://www.biomedcentral.com/1471-244X/12/54/prepub
